# Supplementary material for: Splice-Junction-Based Mapping of Alternative Isoforms in the Human Proteome
Source: Cell Rep. Author manuscript; Available in PMC 2020 Jan 15. (PMC6961840; doi:10.1016/j.celrep.2019.11.026)
Supplement: 2 [file NIHMS1546469-supplement-2.zip › DF1/PXD010154/Liver/Liver_23_HSPG2_DVYMPGPWTLGEGPGQCPAGYPR.pdf]

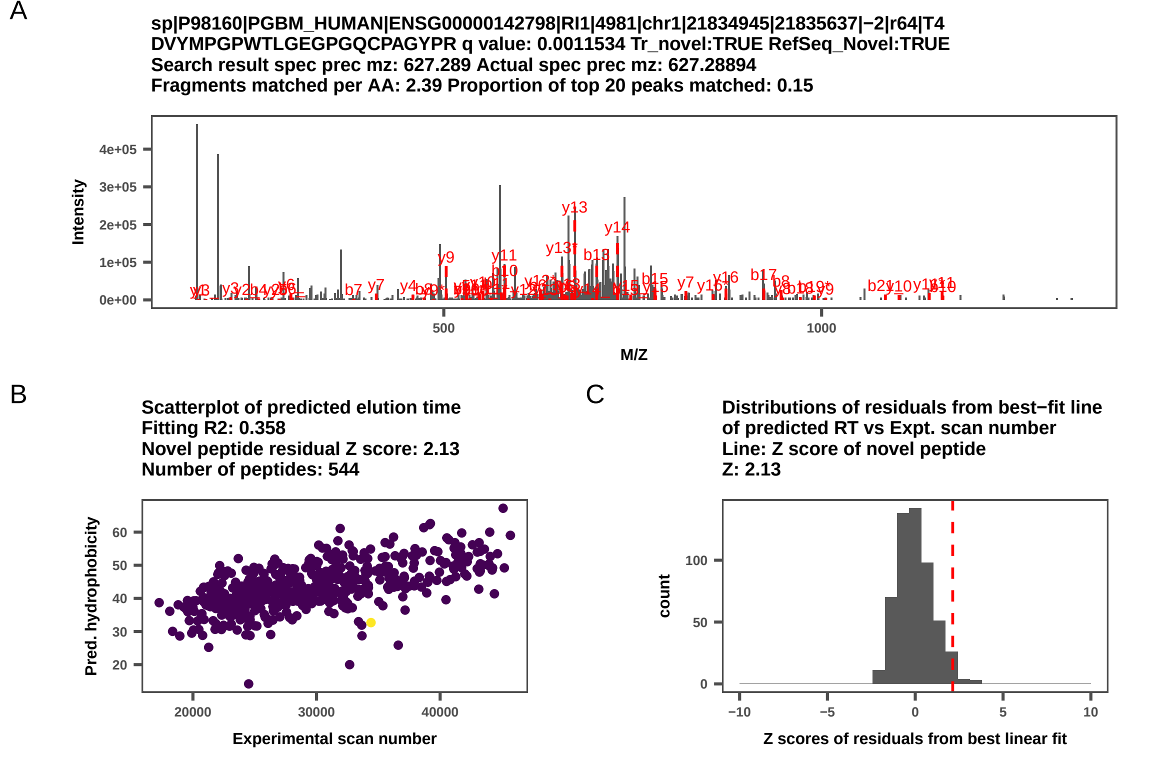

A
sp|P98160|PGBM_HUMAN|ENSG00000142798|RI1|4981|chr1|21834945|21835637|−2|r64|T4
DVYMPGPWTLGEGPGQCPAGYPR q value: 0.0011534 Tr_novel:TRUE RefSeq_Novel:TRUE
Search result spec prec mz: 627.289 Actual spec prec mz: 627.28894
Fragments matched per AA: 2.39 Proportion of top 20 peaks matched: 0.15
Intensity
4e+05
3e+05
2e+05
1e+05
0e+00
y13
y14
y9
y11
b10
b7
y7
b17
M/Z
500
1000
B
Scatterplot of predicted elution time
Fitting R2: 0.358
Novel peptide residual Z score: 2.13
Number of peptides: 544
Pred. hydrophobicity
60
50
40
30
20
20000
30000
40000
Experimental scan number
C
Distributions of residuals from best−fit line
of predicted RT vs Expt. scan number
Line: Z score of novel peptide
Z: 2.13
count
100
50
0
−10
−5
0
5
10
Z scores of residuals from best linear fit
